# Indents

**Published:** 1906-10-15

**Authors:** 


### INDENTS.

☞ **Brief Articles of Technical or Scientific Value will receive notice and comment. Contributions invited.**

**Carbolic Acid in the Preparation of Cavities.** Wiping a cavity with an alcohol-saturated solution of carbolic acid will show very clearly whether all carious tissue has been excavated, and will bring out in relief, so to speak, some crevice that might otherwise possibly be overlooked.—*Dentists Magazine.*

**Hydrogen Dioxid.** Dr. H. W. Hoyt of Rochester, N. Y. says Hydrogen Dioxide in all of its forms is not a germicide, it simply destroys the material on which germ develope. When forced into a sinus it drives the germs into the smaller cavities, but does not kill them. It is a good cleanser of open spaces but should never be used in restricted sinuses.—*Cosmos.*

**Matrix for Pouring Plaster Molds.** Dr. G. E. Truitt in the October *Cosmos*, says, take a strip of tape two inches wide and eleven inches long and dip it into melted wax until saturated and allow it to cool.

When wanted for use, warm slightly and wrap around the impression in the tray to which it will snugly adhere. When the plaster has set it will readily peel off.

**Mixing Amalgam.** Try mixing your amalgam with a stiff steel spatula on glazed paper. Begin as usual with the mortar mix, then transfer to the paper and with the spatula triturate it thoroughly by placing the spatula on the mass and drawing it toward you. You can in this way make a homogeneous mass. Mix in this manner three or four times using a clean place on the paper each time, and it will also clean the amalgam.—H. N. Jackson, *in Review.*

**Amalgam.** I think amalgam may be said to be on the suspect list of filling materials because the personal equation seems to enter more into the making of an amalgam filling than any other filling material. It has poor edge strength. It is brittle. It is an unsatisfactory filling material in those teeth in which there is much overbite; where there are long cusps and deep sulci. The edges give way rapidly under the stress of mastication.—J. E. Nyman, *in Review*]

**Method of Setting Logan Crown.** Prepare the root in the usual manner, and grind the crown to an approximate fit. Mix a small amount of Ascher's Artificial Enamel and place on the base of the crown. Slip over the pin a disk of very thin aluminum or tinfoil and force the crown to place. Remove and lay aside till the ''enamel'' thoroughly hardens, and with suitable disks, well oiled with vaseline, trim to the line on the foil indicating periphery of the root. Remove the foil and set in the usual manner. This gives as near a perfect fit as can be made.—Mark Hayter, *in Review.*

**Dental Art.** In regard to the ideal face, I can not conceive how it is possible to agree upon one of a fixed type, since the ideal of each individual differ from that of another. No two human beings are exactly alike. The color of the hair, the eyes, the teeth, the general form of the face or the complexion differs in some respect from every other individual, but if we have studied art from the artist's point of view sufficiently, and have assimilated what we have studied, we will be able to recognize the form and color of teeth appropriate to each individual case that comes to us, and to restore lost contour in such manner that the result will be harmonious.—J. H. Prothero, *in Review.*

**Construction of Properly-formed Crowns.** Have the band formed and fitted to the root ready for the grinding surface. Warm a bit of impression compound and place it in the band and have the patient grind the teeth then remove the band and compound together and wipe out and dry the inside of the root and of band and compound and fill with melted hard wax and when cool trim off the surplus compound exposing the edge of band about one-half its thickness then carve and groove the grinding surface. This surface is formed by the bite of the opposing teeth so there can be no interfering cusps or planes; the grooves add to the appearance, form avenues of escape and increase the grinding and crushing possibilities. Now mix a little plaster and place it on a paper tablet; fill the grooves and depressions with the plaster to prevent air bubbles. Set the grinding surface of the crown in plaster allowing the plaster to extend a little way up the band. When plaster is set remove the crown and trim plaster almost to the shoulder made by band in the impression. This leaves a flat surface around the impression. Set the small end of a common open-ended sewing-thimble, or a ring of mouldine around the impression and pour it full of fusible metal just as it begins to thicken. Drive this die into a piece of lead for a counter; cut a disk of thin plate (I use 24K about as thin as I can roll it), and swage it between these dies; trim the edges of disk almost to the shoulder, place this cap of disk on crown over compound and burnish the edges down close around band, remove cap, warm band and push out compound and wax burn wax from band and replace cap. It will only go on in the right way and the shoulder stops at the right place. You can attach cap to band with a bit of 20K solder and afterwards put in reinforcement, or the attaching of cap and reinforceing can be done at the same time.

Now, a word as to the re-inforcement. We want a uniform thickness of the grinding surface and do not want the solder to climb the walls. To obtain the one and prevent the other, cut a disk of thin plate and perforate it full of holes with a small punch (this is to prevent air bubbles between the disks), swage this disk between the dies; trim the edges just enough to let this disk drop into the crown; the disk will lay close in the bottom; drop in bits of fluxed solder; flow and all will go right. The disk will act as a uniform carrier and at the same time prevent solder from climbing walls.

File off overlapping edge of cap and polish and the crown is ready to set in the usual way.

To put these principles into a bridge requires an articulator that will give or permit the natural grind of each case. —H. B. Harrell *in Items*.

